# Assessment of maxillary posterior teeth pathologies and its association with maxillary sinus alterations—A Cone Beam Computed Tomography study

**DOI:** 10.4317/jced.62586

**Published:** 2025-12-30

**Authors:** K. B. A. Kader, Ashish Aggarwal, Nitin Upadhyay, Sowmya Gujjar Vishnu Rao, Nupur Agarwal, Ankit Singh Rathore, Jyoti Bisht, Fatima Injela Khan, Shreya Verma, Arifa Naaz

**Affiliations:** 1 Post Graduate Student, Dept. of Oral Medicine & Radiology, Institute of Dental Science, Bareilly, U.P; 2 Professor &amp; Head, Dept. of Oral Medicine & Radiology, Institute of Dental Science, Bareilly, U.P; 3 Professor, Dept. of Oral Medicine & Radiology, Institute of Dental Science, Bareilly, U.P; 4 Senior lecturer, Dept. of Oral Medicine & Radiology, Institute of Dental Science, Bareilly, U.P; 5 Senior lecturer, Dept. of Oral Medicine & Radiology, Institute of dental Science, Bareilly, Uttar Pradesh

## Abstract

**Background:**

Due to the fact that the roots of the maxillary posterior teeth are relatively close to the sinus floor, maxillary sinusitis is frequently caused by odontogenic infections. Mucosal thickness may result from the periapical pathology spreading to the sinuses. This study uses cone-beam computed tomography (CBCT) images to assess the relationship between maxillary sinus mucosal alterations and periapical diseases of maxillary posterior teeth.

**Material and Methods:**

CBCT images were used in this study to assess 302 maxillary posterior teeth with periapical pathologies. Changes in the sinus mucosa were used to measure the proximity the roots and upper border of the lesion to the sinus floor. The CBCT periapical index was used to measure and score the size of the periapical lesion. Analysis was done on the type, pattern, and severity of mucosal thickening, which was considered abnormal if it exceeded 2 mm. Chi-squared tests were used to process the data, and a significance level of p &lt; 0.05 was established.

**Results:**

The study used CBCT to investigate mucosal thickening. Results showed mucosal thickening in 63-71% of cases across various CBCT periapical indexes, with no significant differences among indexes. Significant differences were observed in relation to cortical expansion and destruction, with the thickening being more prevalent in cases with cortical expansion and less in those with destruction.

**Conclusions:**

This study using CBCT reveals significant insights into mucosal thickening, with a prevalence observed in most cases. Cortical expansion increases prevalence, while cortical destruction decreases it. Findings guide effective diagnosis and treatment planning.

## Introduction

Maxillary sinusitis is one of the most prevalent conditions affecting the maxillary sinus. Given the close proximity of the maxillary posterior teeth roots to the sinus floor, infections can easily spread and lead to thickening of the sinus mucosa (1). Allergies or respiratory infections are commonly identified as primary causes of maxillary sinusitis, which affects individuals globally (2). The maxillary sinus (MS) floor, running from the first premolar to the maxillary tuberosity, is often involved in such cases. A notable percentage of maxillary sinusitis cases are odontogenic in origin, owing to the adjacency of the roots of the maxillary posterior teeth to the sinus floor (3 , 4). Periapical infections, even in the absence of a cortical sinus floor perforation, can impact the sinus mucosa by spreading through the bone marrow, blood vessels, and lymphatics. Bacterial toxins and pulpal necrosis products further contribute to inflammation in the MS. When diagnosing maxillary sinus anomalies and periapical alterations, radiographs play a crucial role. The relationship between the MS floor and the roots of the maxillary posterior teeth poses challenges in assessing diffuse discomfort in the posterior maxilla (5). Proper differential diagnosis is essential due to the shared nerve supply between the maxillary teeth and the MS. Approximately 10-12% of maxillary sinusitis cases have an odontogenic origin, making this area highly susceptible to pathogen invasion from the oral cavity or nasal ostium (6 , 7). Odontogenic sinusitis develops by spread of infection, generally of the maxillary tooth, which impairs the Schneiderian membrane. The sinus floor serves as an efficient barrier that stops odontogenic infections from penetrating since it is made of substantial cortical bone. However, in patients whose tooth roots are too near the sinus floor, odontogenic infections may flow into the sinus (8). The primary aim of this study was to evaluate the association between maxillary posterior teeth periapical pathologies and maxillary sinus mucosal changes using cone-beam computed tomography (CBCT). Unlike traditional 2D radiographs, CBCT imaging offers thin CT slices and multiple planes, enabling detailed visualization of both bone and soft tissues, thus serving as a standard diagnostic tool. The study highlights the importance of CBCT in identifying odontogenic maxillary sinusitis due to its lower radiation dose and superior spatial resolution, making it a valuable supplementary diagnostic method.

## Material and Methods

**Figure 1:**
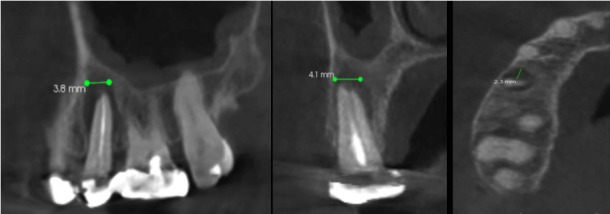
Measurement of the size of periapical lesion on cone-beam computed tomography (CBCT) image. The arrows show (A) Mesiodistal measurement on sagittal section. (B) Buccolingual measurement on coronal section of CBCT. (C) Measurement of depth of periapical lesion on axial section.

This study was conducted among patients who visited the outpatient Department of Oral Medicine and Radiology, Institute of Dental Science, Bareilly. Before conducting the study, ethical clearance was obtained from the institutional ethical board of Institute of Dental Science, Bareilly. The study included 151 CBCT images taken for various clinical indications, which included maxillary CBCT scans displaying fully erupted first and second premolars and molars with fully formed roots, and maxillary posterior teeth with periapical lesions. A total of 302 teeth were observed. Patients with traumatic injuries, sinus issues related to implants or augmentation, or scans not showing complete visualization of the maxillary sinus in coronal and axial views were excluded. The CBCT scans were evaluated by a single investigator. For interpretation of the sinus features and dental pathology, the investigator was instructed by an oral radiologist who had considerable experience in CBCT interpretation. For the calibration and assessment of the intra-examiner reliability, 15 randomly selected scans were evaluated and measured twice by the investigators on 3 different days (mean difference = 0.011 ± 0.004 mm). All the scans showing maxillary sinus mucosal thickening with periapical pathology were obtained using a Kodak Carestream CS 9300 CBCT machine. Imaging protocols included 10 mA (current), 90 kVp (voltage), 90 µm (voxel size), and slice thickness of 75 µm. CBCT images were created in DICOM format and processed using CS 3D software to standardize image placement. A total of 302 maxillary posterior teeth were assessed by multiplanar reconstruction (coronal, sagittal, axial) to measure the periapical lesion size in three sections (Fig. 1).

1

**Figure 2:**
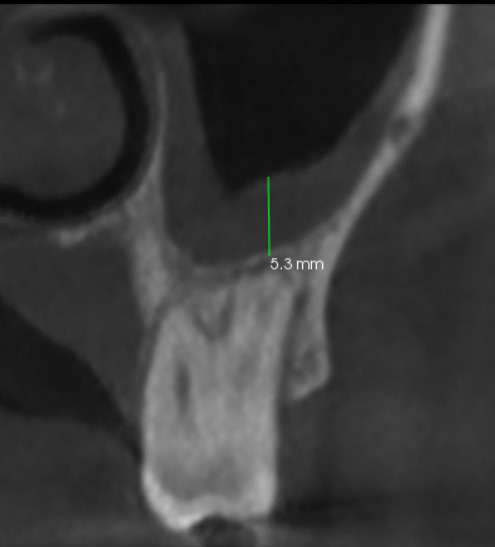
Coronal section showing the measurement of mucosal thickening.

The lesion measurements were scored based on the CBCT-PAI index according to Kaimal V et al., 2023 (1). Scoring Criteria: 0: Intact periapical bone structures 1: Diameter of periapical radiolucency &gt;0.5-1mm 2: Diameter of periapical radiolucency &gt;1-2mm 3: Diameter of periapical radiolucency &gt;2-4mm 4: Diameter of periapical radiolucency &gt;4-8mm 5: Diameter of periapical radiolucency &gt;8mm +E: Expansion of periapical cortical bone +D: Destruction of periapical cortical bone Anatomical Relationship and Measurements: The relationship between maxillary teeth and the sinus was classified into three types index according to Kaimal V et al., 2023 (1): Type I: Space between roots and sinus floor Type II: At least one root in contact with the sinus floor Type III: At least one root entered the sinus floor Lesion and sinus floor relationship was measured and classified index according to Kaimal V et al., 2023 (1): Type I: Lesion extended into the sinus Type II: Lesion was juxtaposed to the sinus floor (0mm) Type III: Distance from top edge of lesion to sinus floor &gt;0 to &lt;2mm Type IV: Distance from top edge of lesion to sinus floor 2mm Mucosal Thickening Classification: Mucosal thickening was classified as generalized or localized and measured in the coronal section (Fig. 2) index according to Kaimal V et al., 2023 (1): Grade I: Normal (0-2mm) Grade II: Moderate (2-10mm) Grade III: Severe (&gt;10mm) All the data were entered into Microsoft Excel and analyzed using SPSS software version 16.0. Chi-square tests and other necessary tests were performed based on data distribution.

2

## Results

The present study investigated the presence of mucosal thickening using cone-beam computed tomography (CBCT) using various indexes and conditions. The study involved 55 premolars (18%) (29 on right side &amp; 26 on left side) and 247 molars (82%) (132 on right side and 115 on the left side). Teeth with periapical lesions were most frequently first and second molars. Mucosal thickening was more frequently observed in relation to teeth with periapical lesion and no significant association was observed between any particular tooth (premolar or molar). Findings revealed mucosal thickening in 63.46% of cases with CBCT periapical index 2, 63.08% with index 3, and 70.59% with index 4, with no significant difference among these indexes. Significant differences were observed in the presence of mucosal thickening in relation to cortical expansion and cortical destruction. Specifically, mucosal thickening was more prevalent in cases with cortical expansion (82.76%) compared to those without (53.76%), and less prevalent in cases with cortical destruction (59.05%) compared to those without (78.26%). Additionally, mucosal thickening varied significantly based on the anatomical position and distance scores (Table 1).

Table 1

The study examined the nature and grade of mucosal thickening, finding that the generalized category was present in 50.3% of cases while the localized category accounted for 49.7% of cases. Additionally, the grades of mucosal thickening were categorized as normal in 15.9% of cases, moderate in 59.6% of cases, and severe in 24.5% of cases (Table 2).

Table 2

The study explored the characteristics of mucosal thickening in relation to cone-beam computed tomography (CBCT) periapical indexes. Findings showed that in CBCT periapical index 1, mucosal thickening was generalized in 51.9% and localized in 48.1% of cases. In CBCT periapical index 2, the generalized category accounted for 46.2% and localized for 53.8% of cases. In CBCT periapical index 3, the generalized category was present in 65.9% and localized in 34.1% of cases. Additionally, the grades of mucosal thickening were moderate in 60.0% and severe in 40.0% of cases for CBCT periapical index 1; moderate in 82.4% and severe in 17.6% for index 2; and moderate in 67.7% and severe in 32.3% for index 3. No significant differences were observed in the characteristics of mucosal thickening across the CBCT periapical indexes (Table 3).

Table 3

## Discussion

The present study investigated the presence of mucosal thickening using cone-beam computed tomography (CBCT) and explored various indexes and conditions to provide a comprehensive understanding of this phenomenon. Our findings indicated mucosal thickening in a significant proportion of cases, with the highest prevalence observed in CBCT periapical index 4 (70.59%), followed closely by indexes 2 (63.46%) and 3 (63.08%). This demonstrates a consistent presence of mucosal thickening across different CBCT indexes (1 , 2). Interestingly, no significant differences were observed among these indexes, suggesting that mucosal thickening is a common occurrence regardless of the specific index used. However, significant differences were identified in the presence of mucosal thickening concerning cortical expansion and cortical destruction. Specifically, mucosal thickening was more prevalent in cases with cortical expansion (82.76%) compared to those without (53.76%). Conversely, mucosal thickening was less prevalent in cases with cortical destruction (59.05%) compared to those without (78.26%). These findings highlight the influence of cortical changes on the prevalence of mucosal thickening. Additionally, our study examined the nature and grade of mucosal thickening, revealing that generalized thickening was present in 50.3% of cases, while localized thickening accounted for 49.7%. This near-equal distribution emphasizes the variability in the presentation of mucosal thickening. Furthermore, the grades of mucosal thickening were categorized as normal in 15.9% of cases, moderate in 59.6% of cases, and severe in 24.5%. These results underscore the diverse severity of mucosal thickening observed in our study population. The characteristics of mucosal thickening in relation to CBCT periapical indexes were also explored, with findings indicating that mucosal thickening varied across different indexes. For instance, in CBCT periapical index 1, mucosal thickening was generalized in 51.9% of cases and localized in 48.1% of cases. In CBCT periapical index 2, the generalized category accounted for 46.2% and localized for 53.8% of cases, while in CBCT periapical index 3, the generalized category was present in 65.9% and localized in 34.1% of cases. Additionally, the grades of mucosal thickening differed across indexes, with moderate and severe grades being the most prevalent. These findings contribute to a growing body of evidence on the prevalence and characteristics of mucosal thickening, providing valuable insights for clinical practice (16). The variability in mucosal thickening based on cortical conditions, anatomical position, and distance scores underscores the need for careful assessment and individualized treatment planning. Further research is warranted to explore the underlying mechanisms and potential clinical implications of these findings. By comparing your study's findings with previous literature, it becomes evident that mucosal thickening is a prevalent condition associated with various dental and sinus conditions. For instance, Shanbhag et al. reported a 60% prevalence of mucosal thickening among 243 patients, with teeth having periapical lesions being 9.75 times more likely to show mucosal thickening (2). Lu et al. found that more than 80% of 88 teeth with apical periodontitis in maxillary posterior teeth exhibited mucosal thickening (8). Kaimal VG's study showed that 93.4% of participants had mucosal thickening, aligning with our findings (1). Maxillary sinusitis symptoms include headache, head heaviness upon postural changes, nasal congestion, rhinorrhea, and/or foul odor and taste. In our study, 19.8% of participants had a history of sinusitis, with 95% showing mucosal thickening. Most cases showed localized mucosal thickening (52%), while generalized thickening was observed in 43% of participants with a history of sinusitis. These results indicate the potential influence of sinusitis on mucosal thickening. Infections in upper premolars and molars, whether periapical or periodontal, can spread into the maxillary sinus (MS) and cause sinusitis. Conditions such as periapical infection, root canal treatment, and the close relationship between maxillary teeth and the sinus can predispose individuals to mucosal thickening in the MS. After pulp necrosis, bacterial enzymes and toxins promote tissue breakdown in the periapical bone, allowing infections to spread to the MS and potentially cause mucosal irritation. Several authors have correlated the size of periapical lesions and mucosal thickening. For example, Lu et al. and Vallo et al. reported that the prevalence of mucosal thickening increased with the size of the lesion [8,10). Goller-Bulut et al. found that mucosal thickening increased with the severity of apical periodontitis among 205 patients with 410 exposed MS (11). In our study, 100% of participants with a PAI score of 4 had sinus mucosa thickening, while 93% with a PAI score of 3 showed mucosal thickening, indicating a higher prevalence of mucosal thickening with larger periapical lesions, though the difference was not statistically significant. Cortical bone expansion and destruction are critical factors in mucosal thickening. In our study, 88.2% of teeth with cortical bone expansion and 100% of teeth with cortical bone destruction exhibited mucosal thickening. This relationship emphasizes the impact of cortical changes on the prevalence of mucosal thickening. Literature suggests that the palatal root of the maxillary first molar often penetrates the sinus, while the mesiobuccal root of the second molar is adjacent to the sinus, and premolar roots rarely intrude into the sinus cavity. This anatomical relationship poses various risks, especially during surgical procedures like tooth extraction, implant placement, or endodontic or orthodontic treatments. Our study found that 100% of participants with type II and III anatomical positions of the root relative to the sinus showed mucosal thickening. Conversely, a retrospective study by Estrela concluded that the anatomical relationship alone does not affect MS mucosal thickening (7). The close proximity of periapical lesions to the MS may be a potential factor in sinus mucosal thickening. The highest number of abnormalities were observed when the top edge of the periapical lesion extended into the sinus floor, indicating the effect of lesion proximity on the sinus mucosa. This finding is consistent with Nunes et al., who revealed the highest number of abnormalities associated with teeth whose apical lesions were directly adjacent to the sinus floor (3). However, Rege et al. found no significant relation between lesion proximity and sinus mucosa changes in a study of 1,113 patients. Similarly, Lu et al. reported no association between lesion proximity and sinus abnormalities in 508 exposed sinuses (8). The study examines mucosal thickening, classifying it as generalized or localized, flat or polypoidal, and moderate or severe. Among participants, 62.3% had localized thickening. PAI scores II and III displayed localized thickening in 62.5% and 77.3% of cases, respectively, while PAI score IV showed generalized thickening (70.6%). Most cases exhibited flat thickening (65.1%) rather than polypoidal (28.3%). Moderate thickening was predominant (58.5%), with severe cases being less common (37%). The severity of mucosal thickening was influenced by lesion size and proximity to the sinus floor, but not by age. Moderate thickening was more prevalent in anatomical position types I and II, while severe thickening was higher in type III, indicating an increase in severity with closer proximity to the sinus floor. This study underscores the critical role of CBCT in evaluating mucosal thickening, particularly in relation to cortical changes and anatomical positioning, providing valuable insights for effective diagnosis and treatment planning in clinical practice. The study evaluated the effect of lesion size and the spatial relationship of the root and lesion edge to the sinus floor, though the periodontal status of the teeth, which could cause sinus mucosal changes, was not considered.

## Conclusions

The study using CBCT provides significant insights into the prevalence and characteristics of mucosal thickening. Mucosal thickening was observed in most cases, with no notable differences among CBCT periapical indexes 2, 3, and 4. However, cortical expansion significantly increased the prevalence of mucosal thickening, while cortical destruction decreased it. This highlights the importance of considering cortical changes when assessing mucosal thickening. The study categorized mucosal thickening into generalized and localized types, with nearly equal distribution between them. Additionally, the severity of mucosal thickening varied, with cases categorized as moderate being the most predominant, followed by severe and normal. Overall, the study highlights the critical role of CBCT in evaluating mucosal thickening, particularly in relation to cortical changes and anatomical positioning. These findings provide valuable insights for clinicians, aiding in accurate diagnosis and effective treatment planning. This research contributes to a better understanding of the complex relationship between periapical lesions and sinus mucosal thickening, emphasizing the need for comprehensive assessment using CBCT.

## Figures and Tables

**Table 1:** Comparison of CBCT-PAI, cortical expansion and destruction, anatomical position of root to sinus floor, and distance from the top edge of lesion to sinus floor with sinus MT using the chi-squared test.

Variable	Category	Mucosal Thickening Present		Mucosal Thickening Absent		P-Value
		Number	%	Number	%	
CBCT-PAI	PAI 2	33	63.46	19	36.54	0.732#
	PAI 3	41	63.08	24	36.92	
	PAI 4	24	70.59	10	29.41	
Cortical Expansion	Present	48	82.76	10	17.24	0.000*
	Absent	50	53.76	43	46.24	
Cortical Destruction	Present	62	59.05	43	40.95	0.023*
	Absent	36	78.26	10	21.74	
Anatomical Position	Type I	44	66.67	22	33.33	0.015*
	Type II	33	78.57	9	21.43	
	Type III	21	48.84	22	51.16	
Distance	Score 1	33	64.7	18	35.3	0.125#
	Score 2	53	67.9	25	32.1	
	Score 3	6	40.0	9	60.0	
	Score 4	6	85.7	1	14.3	

Abbreviations: CBCT-PAI, cone-beam computed tomography-periapical index; Statistically significant.

**Table 2:** Characteristics of mucosal thickening of maxillary sinus among study subjects.

Variable	Category	Number	%
Nature of thickening	Generalized	76	50.3
	Localized	75	49.7
Grade	Normal	24	15.9
	Moderate	90	59.6
	Severe	37	24.5

2

**Table 3:** Association between CBCT-PAI and characteristics of mucosal thickening using chi-squared test.

	Type				P-Value
CBCT-PAI	Generalized		Localized		
	n	%	n	%	
PAI 2	27	51.9	25	48.1	0.123#
PAI 3	30	46.2	35	53.8	
PAI 4	29	65.9	15	34.1	
	Severity/grade				
	Moderate		Severe		
PAI 2	27	60.0	18	40.0	0.050#
PAI 3	42	82.4	9	17.6	
PAI 4	21	67.7	10	32.3	

3

## Data Availability

The datasets used and/or analyzed during the current study are available from the corresponding author.
